# Differential Regulation of MAPK Phosphorylation in the Dorsal Hippocampus in Response to Prolonged Morphine Withdrawal-Induced Depressive-Like Symptoms in Mice

**DOI:** 10.1371/journal.pone.0066111

**Published:** 2013-06-18

**Authors:** Wei Jia, Rui Liu, Jianguo Shi, Bin Wu, Wei Dang, Ying Du, Qiong Zhou, Jianhua Wang, Rui Zhang

**Affiliations:** 1 Methadone Maintenance Treatment Clinic, Xi’an Mental Health Center, Xi’an, China; 2 Department of Geriatrics, Tangdu Hospital, Fourth Military Medical University, Xi’an, China; 3 Department of Neurology, Tangdu Hospital, Fourth Military Medical University, Xi’an, China; Temple University, United States of America

## Abstract

Depression is one of the most frequent neuropsychiatric comorbidities associated with opiate addiction. Mitogen activated protein kinase (MAPK) and MAPK phosphatase (MKP) are involved in drug addiction and depression. However, the potential role of MAPK and MKP in depression caused by morphine withdrawal remains unclear. We utilized a mouse model of repeated morphine administration to examine the molecular mechanisms that contribute to prolonged withdrawal induced depressive-like behaviors. Depressive-like behaviors were significant at 1 week after withdrawal and worsened over time. Phospho-ERK (extracellular signal-regulated protein kinase) was decreased and MKP-1 was elevated in the hippocampus, and JNK (c-Jun N-terminal protein kinase), p38 (p38 protein kinase) and MKP-3 were unaffected. A pharmacological blockade of MKP-1 by intra-hippocampal sanguinarine (SA) infusion prevented the development of depressive-like behaviors and resulted in relatively normal levels of MKP-1 and phospho-ERK after withdrawal. Our findings support the association between hippocampal MAPK phosphorylation and prolonged morphine withdrawal-induced depression, and emphasize the MKP-1 as an negative regulator of the ERK phosphorylation that contributes to depression.

## Introduction

Drug addiction is widely accepted as a relapsing brain disease [1]. The primary goal of drug addiction treatment is to maintain abstinence. In chronic opiate users, abrupt withdrawal produces a well-characterized aversive emotional state along with physical signs that typically last for a few days. Once this acute period has passed, individuals begin to experience more complex and long-lasting depressive-like symptoms [2]. Treatment of depressive symptoms that following opiate addiction is an important factor affecting the long-term outcomes of drug addiction treatment [3].

The hippocampus plays critical roles in drug-associated memory, addictive behaviors and mediation of the cognitive aspects of depression [4], [5]. The hippocampus is involved in the consolidation of long-term memory in addiction [6]. Addictive drugs can disrupt neurogenesis in the adult hippocampus [7]. Consistent with a reduction in the hippocampal volume in individuals with major depression, brain imaging and postmortem studies demonstrat of apoptosis and atrophy of the hippocampal pyramidal neurons [8]–[10]. Chronic antidepressant treatment notably promotes neurogenesis in the adult hippocampus, and this cellular response is required for antidepressants to ameliorate depressive-like symptoms in certain animal models of depression [11]. However, the underlying molecular mechanisms that regulate the structural and physiological alterations of the hippocampus during episodes of opioid withdrawal-induced depression remain unclear.

The mitogen activated protein kinase (MAPK) pathway is a major signaling system that regulates cellular responses, including neuronal plasticity, function, survival and apoptosis [12]–[14]. The MAPK are activated by phosphorylation and selectively inactivated by MAP kinase phosphatase (MKP) that are either induced or activated by MAPK [15]–[17]. MKP-1 and MKP-3 are selective for extracellular signal-regulated protein kinase (ERK), c-Jun N-terminal protein kinase (JNK) and p38 protein kinase (p38) [16]. Evidence implicates changes in MAPK and MKP activity in both drug-induced behavioral changes and aberrant emotional states. Repeated morphine injection increases phospho-ERK levels in the mouse hippocampus [18]. The administration of cocaine, morphine and methamphetamine (METH) both *in vivo* and *in vitro* activates MKP-1 and/or MKP-3 in several cortices, the caudate putamen, the nucleus accumbens and the hippocampus [19]–[21]. The activation of the MAPK cascade and the regulatory effects of MKP play a critical role in the pathophysiology of depression, and alteration of the activity of MAPK or MKP may prove useful for the treatment of the disease. The postmortem and preclinical studies identify MKP-1 as a negative regulator in major depression pathophysiology [22].

The association between drug withdrawal and depressive-like symptoms is well documented, but research has not fully explained the exact molecular mechanisms, particularly the interaction between MAPK and MKP in the hippocampus. In this study, we utilized a mouse model of repeated morphine administration to examine the consequences of prolonged withdrawal-induced depressive-like behaviors. We quantified the withdrawal symptoms and depressive-like behaviors and analyzed the levels of phospho-ERK, phospho-JNK, phospho-p38, MKP-1 and MKP-3 in the hippocampus of mice over the course of morphine administration and after a prolonged period of withdrawal and pharmacological blockade of MKP-1 by intra-hippocampal sanguinarine (SA) infusion.

## Materials and Methods

### Animals

A total number of 170 male C57BL/6J mice (8 weeks old, 22–28 g) were housed in groups of four per cage (20±2°C, 50±5% humidity) under a 12/12 h light/dark cycle (lights on at 07∶00) with food and water *ad libitum*. The mice were acclimated to the housing conditions for 1 week before experimental manipulations. All animal protocols were approved by the Institutional Animal Care and Use Committee of the Fourth Millitary University, and every effort was made to minimize both the suffering of the animals and the number of animals utilized.

### Drugs and Antibodies

Morphine HCl (The First Pharmaceutical Factory of Shenyang, China) was dissolved in 0.9% sodium chloride. The sanguinarine chloride (SA, a selective inhibitor of MKP-1) and dimethylsulfoxide (DMSO) were purchased from Sigma-Aldrich (St. Louis, MO, USA). SA was dissolved in DMSO to achieve the required dose. The primary antibodies against phospho-ERK (Thr202/Tyr204), phospho-JNK (Thr183/Tyr185), phospho-p38 (Thr180), ERK, JNK, and p38 were purchased from Cell Signaling Technology (Beverly, MA, USA). The primary antibodies against MKP-1, MKP-3, β-actin, and horseradish peroxidase-conjugated anti-rabbit or anti-goat secondary antibodies were purchased from Santa Cruz Biotechnology (Santa Cruz, CA, US).

### Morphine Treatment Protocols

Repeated morphine administration paradigm was performed by using a previously established procedure with a minor modification [23]. Briefly, mice received escalating doses of morphine (10–100 mg/kg, i.p., twice daily at 09∶00 and 16∶00) or saline administered i.p. for 8 consecutive days. Withdrawal behaviors were observed (irritation and diarrhea) and quantified (teeth chattering, wet-dog shaking, grooming, fore paw tremor, rearing and jumping) by an observer blind to the treatments at 2, 12, 24, 48 and 72 h after the last injection. Each behavioral observation session lasted 20 min. For each mouse, the score on the withdrawal syndrome rating scale was the score of observed signs (on a scale of 0–3) plus the score of the counted behaviors (multiplied by weighting factors) that occurred during the observation period (see Table S1) [24]. The body weight was recorded at 24, 48 and 72 h after the last injection. Depressive-like and social behaviors were examined on day 15 and day 36 (1 and 4 weeks of withdrawal) after the last injection of either morphine or saline control.

### Stereotaxic Surgery and SA Microinjections

Mice were anaesthetized with 4% chloral hydrate (120 ml/kg) and 4% urethane (1000 ml/kg) and placed in a stereotaxic device (RWD Life Science Co., Ltd, Shenzhen, China). Bilateral stainless steel guide cannulas were inserted at the following Bregma coordinates: anterioposterior (AP) = −1.8 mm, mediolateral (ML) = −1.8 mm, dorsoventral (DV) = −1.8 mm [25], [26]. Mice were left undisturbed for 5 days to recover from the surgery. Intra-hippocampal SA microinjections were performed through a stainless steel cannula of outside tip diameter 0.28 mm, connected to a 0.5 µl Hamilton microsyringe. A volume of 5.0 µg/0.2 µl per side of SA [27] or the same volume of vehicle (DMSO) was injected bilaterally into the dorsal hippocampus within 2 min, and the needles were left in place for an additional 60 s to facilitate the diffusion of the drug. The microinjections were performed once daily (at 8∶00 a.m.) for 8 consecutive days.

### Behavioral Testing

To test the depressive-like and social behaviors of mice, the animals underwent sequential open-field, social interaction and tail suspension tests.

#### Open-field test

The mice were placed in the center of a black square arena (45×45×40 cm, Panlab, Barcelona, Spain). During a 30 min session, the animals were scored for the distance traveled and time spent in the center of the box (25 cm^2^ area). The mouse behavior was recorded with an automated video-tracking and analysis system (Smart Ver2.5, Panlab, Barcelona, Spain).

#### Social interaction test

The following procedures were adapted from Miyakawa et al [28]. Pairs of mice from the same treatment condition, but from different home cages, were placed in the same arena for 10 min. Social interaction (i.e., the time in seconds the mice engaged in sniffing, following, allogrooming, biting and mounting with partners) was recorded and analyzed by four inter-observers (kappa test for inter-observer variation, κ = 0.85).

#### Tail suspension test (TST)

The mice were suspended on the edge of a shelf 60 cm above a table top by adhesive tape placed approximately 1 cm from the tip of the tail. The duration of immobility was automatically recorded for a period of 6 min and analyzed by four inter-observers.

### Western Blots

Mice were sacrificed by decapitation, and the brains were quickly removed, frozen on dry ice and stored at −80°C. The hippocampus was dissected and homogenized in 300 µl ice-cold extraction buffer (50 mM Tris–HCl pH 7.5, 50 mM NaCl, 5 mM EDTA, 10 mM EGTA, 2 mM sodium pyrophosphate, 4 mM paranitrophenylphosphate, 1 mM sodium orthovanadate, 1 mM phenylmethylsulfonyl ﬂuoride, 2 µg/ml aprotinin, 2 µg/ml leupeptin and 2 µg/ml pepstatin). The homogenate was incubated on ice for 20 min and centrifuged at 13,000 *g* for 20 min at 4°C [29]. The supernatant was collected for a protein assay utilizing the Bradford method (Bio-Rad, Hercules, US). The samples were denatured at 95°C for 5 min, separated by 12% SDS-PAGE and transferred to PVDF membranes. The membranes were blocked with 5% non-fat milk for 1 h at 22°C and incubated with primary antibodies against phospho-ERK, phospho-JNK, phospho-p38, MKP-1 or MKP-3 at 1∶1000 dilutions kept overnight at 4°C. The membranes were incubated with a HRP-conjugated secondary antibody for 1 h at room temperature and developed with enhanced chemiluminescence. β-actin (1∶5000) was utilized to normalize the levels of MKP-1 and MKP-3. The immunoreactive protein bands were quantified by densitometry using QuantityOne Ver. 4.6.2 (Bio-Rad, Hercules, US).

### Experiment 1

Mice were randomly divided into two groups: a morphine-exposed group (n = 16) and a saline control group (n = 16). Within each group, 10 mice underwent behavioral testing and 8 mice underwent protein expression determination. After 8 days of morphine administration, mice were subjected to 4 weeks of withdrawal. Somatic withdrawal behaviors were observed and quantified at 2, 12, 24, 48 and 72 h after the last morphine injection. The depressive-like and social behaviors of mice were determined by open-field, social interaction and tail suspension tests after 1 and 4 weeks of withdrawal. Mice were sacrificed at 2 h, 1 and 4 weeks of morphine withdrawal. The protein expression in hippocampus was determined by western blots.

### Experiment 2

Experiment 2 was initiated when body weights of mice had been stabilized for one week. A total of 48 mice were randomly assigned into three groups (n = 16 of each group): (i) Ad libtum feeding (AF) group, which were allowed free access to food pellet; (ii) Restricted feeding (RF) group, which food was restricted to 1.5 g per mouse each day for 8 consecutive days. Once the restricted feeding session passed, those RF mice were allowed free access to food pellet again until the end of the study. (iii) Morphine-treated (MT) group, which received escalating doses of morphine (10–100 mg/kg, i.p., twice daily) for 8 consecutive days. MT group were allowed free access to food pellet throughout the experiment. All mice were freely provided tap water. Body weights were recorded daily. Half of the subjects in the AF, RF and MT groups were killed at day 8, while the other half of the subjects were killed at day 15. The impact of body weight change on hippocampal ERK, MKP-1 and MKP-3 expression was evaluated by western blot.

### Experiment 3

Experiment 3 was performed on a new set of mice that were divided into 5 groups (n = 18 per group): naïve, saline/DMSO, saline/SA, morphine/DMSO, morphine/SA. Within each group, 10 mice underwent behavioral testing and 8 mice underwent protein expression determination. Naïve mice were not subjected to stereotaxic surgery or any drug administration. After recovered from the stereotaxic surgery, mice were treated with different combination of intra-hippocampal SA infusion and morphine i.p. injection. Next, mice were subjected to 4 weeks of withdrawal. Somatic withdrawal signs and depressive-like behaviors of mice were determined during the withdrawal period. Mice that underwent protein expression determination did not undergo any behavioral tests. These mice were sacrificed at 2 h, 1 and 4 weeks of morphine withdrawal. The protein expression in the hippocampus was determined by western blots.

### Statistical Analysis

Data analysis was performed with GraphPad Prism (Ver. 5.0, GraphPad Inc., US). The significant differences in body weight, withdrawal score, depressive-like behaviors and protein changes were determined with a two-way analysis of variance (ANOVA) with independent or repeated measures. Bonferroni post hoc analysis were utilized to reveal difference between groups. The levels of phosphorylated proteins are expressed as the ratio to the total protein. For MKP-1 and MKP-3 expression, β-actin was used to normalize data. All values represent the mean ± standard error of the mean (SEM). Statistical significance was defined as p<0.05.

## Results

### Experiment 1

#### Repeated morphine treatment induces physical signs that decreases during withdrawal

**Figure 1:**
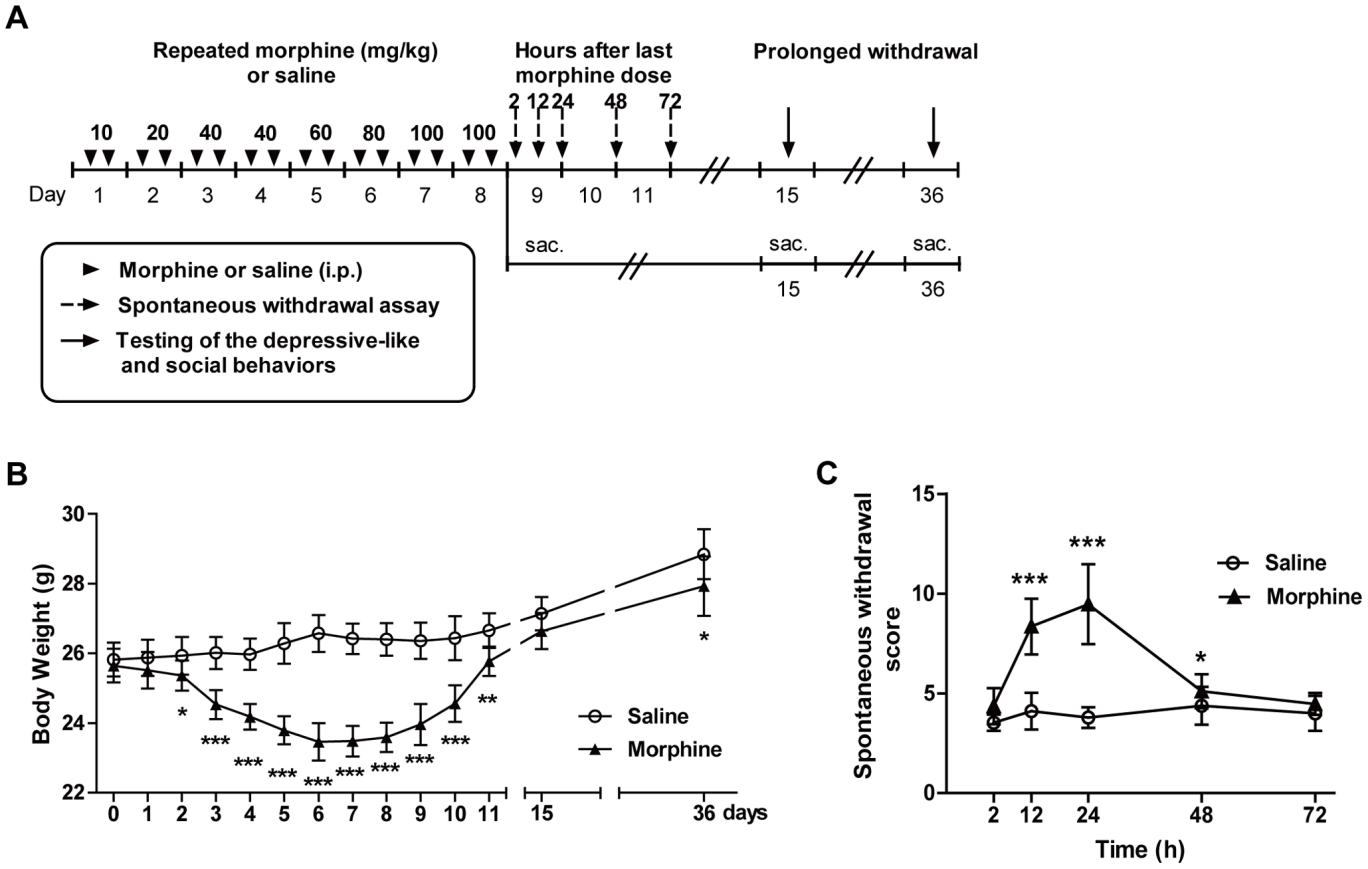
Body weight change and withdrawal scores in the experiment 1. (A) Timeline of the experiment 1. The duration of each stage (number of days) is shown under the axis. Black triangles indicate escalating doses of morphine or saline injected twice daily for 8 consecutive days. Dash arrows indicate the observation of the withdrawal behaviors at different time points after the last morphine administration. Solid black arrows indicate the behavioral testing at two withdrawal time points during the study. “Sac.” indicates that mice were sacrificed. (B) The influence of repeated morphine injection and prolonged withdrawal on body weight during the experimental period. (C) Withdrawal scores following the last dose of morphine injection. The data are expressed as the mean ± SEM. Significance relative to control is indicated by *p<0.05; **p<0.01; ***p<0.0001.

The timeline of the experiment 1 is presented in Fig. 1A. The body weight curve is shown in Fig. 1B for the mice under morphine withdrawal for 4 weeks. The mice either underwent behavioral testing or protein assessment. A two-way ANOVA indicated that the morphine treatment reduced body weight over time (morphine: F_1, 252_ = 659.9, p<0.0001; time: F_13, 252_ = 67.33, p<0.0001; interaction: F_13, 252_ = 19.94, p<0.0001). This decrease was significant beginning at the 2nd day of injection (p<0.05) and persisting for 3 days after the last morphine injection on day 8 (p<0.01). After 1 week of withdrawal (at day 15), the body weight of the morphine-treated mice were similar to the saline-treated mice (p = 0.21), but the weight gain of the morphine-treated mice was significantly less than the saline treated mice after 4 weeks of abstinence (at day 36, p<0.05).

We monitored the development of physical signs for 3 days after morphine withdrawal. Morphine withdrawal had significant effects on the global behavioral score (morphine: F_1, 90_ = 133.1, p<0.0001; time: F_4, 90_ = 26.99, p<0.0001) (Fig. 1C). Two hours after the last morphine injection, the mice did not exhibit obvious withdrawal symptoms compared to the saline group (p = 0.44). The withdrawal induced severe behavioral signs that peaked approximately 24 h (p<0.0001) and declined gradually within 72 h (p = 1.09). Therefore, the physical signs induced by morphine withdrawal clearly attenuates as withdrawal unfolds over time (morphine-time interaction: F_4, 90_ = 26.91, p<0.0001).

#### Depressive-like behaviors develop during prolonged withdrawal

**Figure 2:**
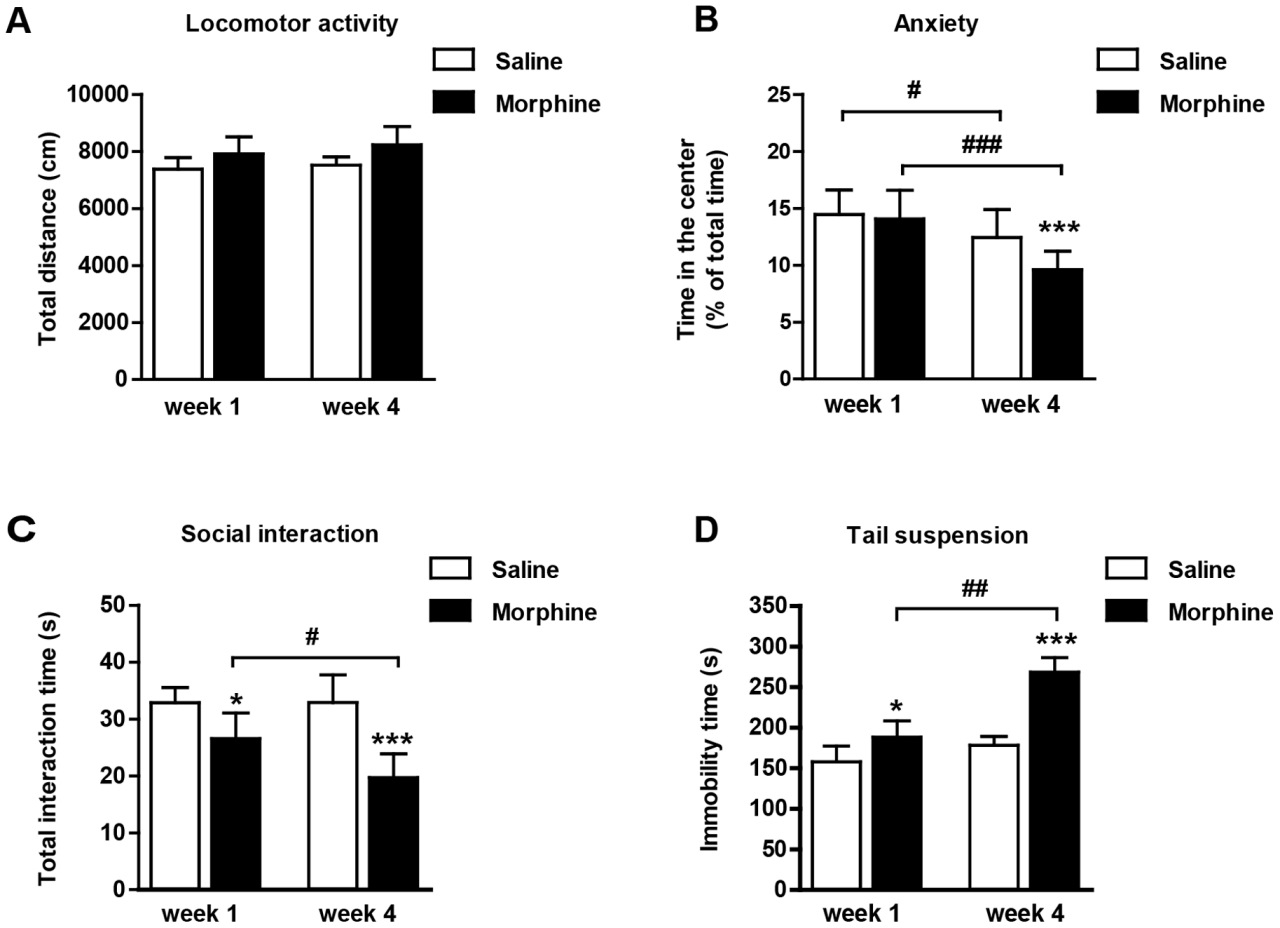
Depressive-like behaviors develop during the morphine withdrawal period. (A) Locomotor activity was not influenced by the previous morphine exposure after 1 or 4 weeks of withdrawal. (B) The morphine treated mice spent less time in the center of the arena after 4 weeks of withdrawal. (C) Social behaviors decreased significantly in morphine-treated mice after the 1-week or 4-week withdrawal period. The 4-week morphine abstinent mice interacted less than the 1-week morphine abstinent group. (D). Immobility in tail suspension test increased significantly in morphine-treated mice during the withdrawal period. The data are expressed as the mean ± SEM. Significance from saline control is indicated as *p<0.05; **p<0.01; ***p<0.0001. Comparison between 1- and 4-week withdrawal groups within each treatment condition is indicated by ^#^p<0.05; ^##^p<0.01; ^###^p<0.0001.

The total distance traveled in the open-field arena was not influenced by previous morphine treatment (F_1, 18_ = 0.23, p = 0.64) or the duration of withdrawal (F_1, 18_ = 0.89, p = 0.35) (Fig. 2A). The saline- and morphine-treated animals spent different percentages of time in the center of the arena (F_1, 18_ = 46.62, p<0.0001) (Fig. 2B). Theeffect of the withdrawal time in morphine-exposed mice was significant (F_1, 18_ = 118.01, p<0.0001). A strong interaction was detected between these factors (F_1, 18_ = 15.74, p<0.0001). Both the saline- and morphine-exposed mice spent less time in the center at the 4-week time point compared to the 1-week time point (p<0.05 and p<0.0001, respectively). Moreover, the morphine-exposed mice spent significantly less time in the center at the 4-week time point compared with the saline controls (p<0.0001).

The total mice social interaction time was affected by both morphine exposure (F_1, 18_ = 70.96, p<0.0001) and duration of withdrawal (F_1, 18_ = 6.42, p<0.05) (Fig. 2C). Moreover, a significant interaction was detected between these factors (F_1, 18_ = 7.81, p<0.05). The Bonferroni post hoc analysis revealed less social interaction in the morphine-treated mice than the saline controls after 1 week (p<0.05) and 4 weeks (p<0.0001) of morphine withdrawal. The social interactions in the morphine-treated mice deceased at 4 weeks compared to 1-week after withdrawal (p<0.05).

A main effect was detected between the treatment (F_1, 18_ = 108.51, p<0.0001) and time (F_1, 18_ = 93.87, p<0.0001) in the TST (Fig. 2D). Moreover, a significant interaction was detected between these factors (F_1, 18_ = 33.38, p<0.0001). Repeated morphine exposure increased immobility at both the 1-week and 4-week time points compared with their saline-treated counterparts (p<0.05 and p<0.0001, respectively). Moreover, the morphine-withdrawal mice exhibited significantly increased immobility times at week 4 compared to week 1 (p<0.01).

#### Regulation of ERK, JNK, p38, MKP-1 and MKP-3 in the mouse hippocampus after repeated morphine treatment and prolonged withdrawal

**Figure 3:**
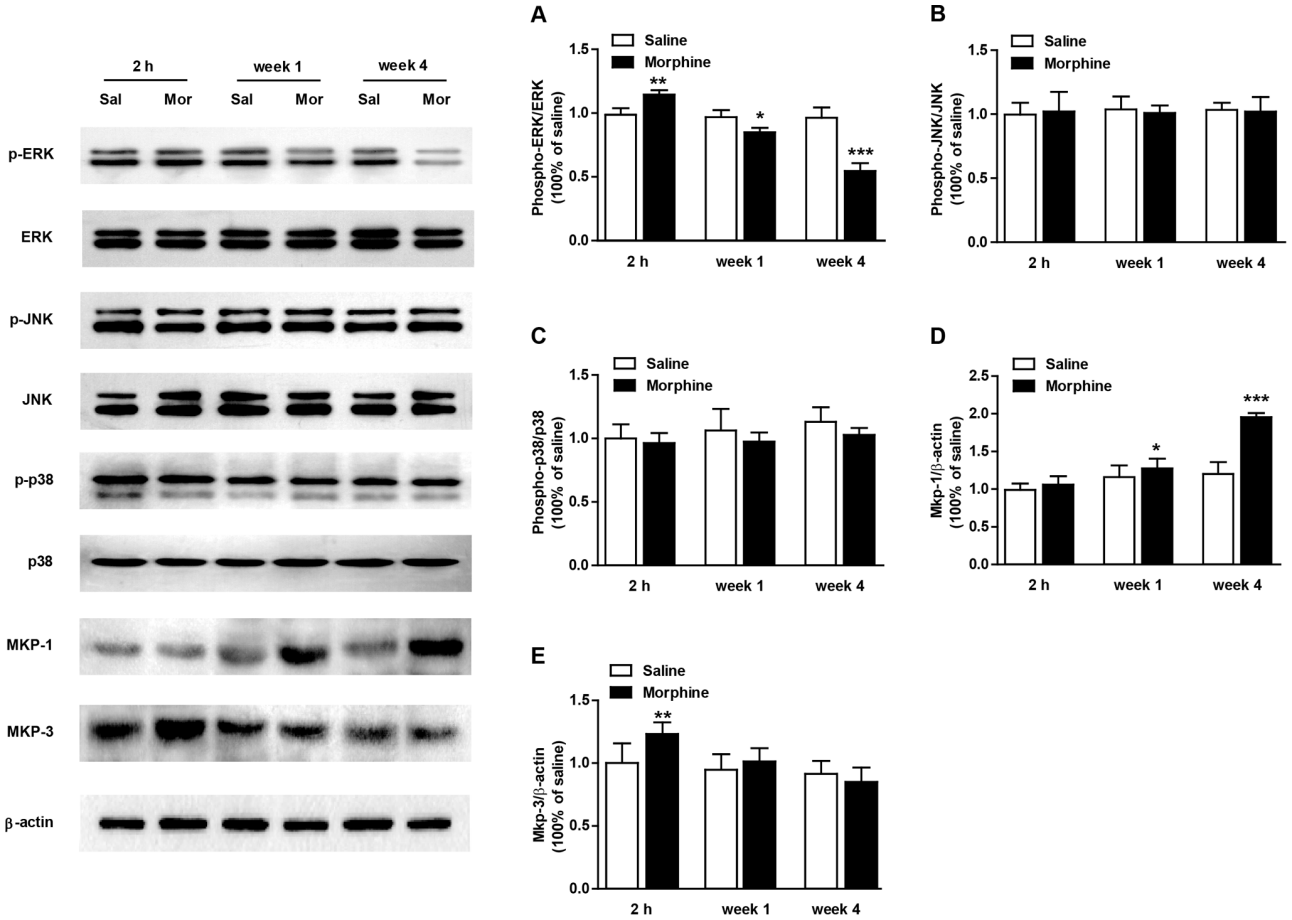
Expression of ERK, JNK, p38, MKP-1 and MKP-3 in the hippocampus in repeated morphine (n = 6) or saline (n = 6) treated mice. Western blots were performed on a separate cohort of animals which did not undergo behavioral testing. Ratios of the phospho-protein relative to total protein levels were analyzed. Data are expressed as the mean ± SEM relative to saline controls that were set as 1. The β-actin was utilized as a loading control. *p<0.05; **p<0.01; ***p<0.0001 compared with the saline group.

The phospho-ERK expression in the hippocampus was affected by both morphine exposure (F_1, 30_ = 5.30, p<0.05) and duration of withdrawal (F_2, 30_ = 27.71, p<0.0001) (Fig. 3A). A significant interaction was detected between the two factors (F_2, 30_ = 21.98, p<0.0001). The Bonferroni post hoc analysis revealed a significant increase in phospho-ERK in morphine-treated mice compared to saline controls at the 2-h time point (p<0.01). After 1 week (p<0.05) and 4 weeks (p<0.0001) of morphine withdrawal, phospho-ERK levels clearly decreased compared with the saline controls. Neither morphine exposure (phospho-JNK: F_1, 30_ = 0.08, p = 0.77; phospho-p38: F_1, 30_ = 0.21, p = 0.65) nor withdrawal time (phospho-JNK: F_2, 30_ = 0.53, p = 0.59; phospho-p38: F_2, 30_ = 0.06, p = 0.94) affected JNK or p38 phosphorylation (Fig. 3B, C).

A two-way ANOVA detected significant effects of morphine exposure (F_1, 30_ = 24.39, p<0.0001) and withdrawal time (F_2, 30_ = 6.53, p<0.01) as well as an interaction (F_2, 30_ = 4.85, p<0.05)with the MKP-1 expression in morphine-exposed mice (Fig. 3D). Notably, the post hoc test revealed increased MKP-1 levels after 1 and 4 weeks of morphine withdrawal (p<0.05, p<0.0001, respectively), with no difference in mice 2 h after receiving the last morphine injection (p = 0.65). MKP-3 expression was affected only by the withdrawal time (F_2, 30_ = 3.93, p<0.05),not by morphine exposure (F_1, 30_ = 3.75, p = 0.063) (Fig. 3E).

### Experiment 2

#### Hippocampal ERK, MKP-1 and MKP-3 are not affected by metabolic changes resulting from weight loss

**Figure 4:**
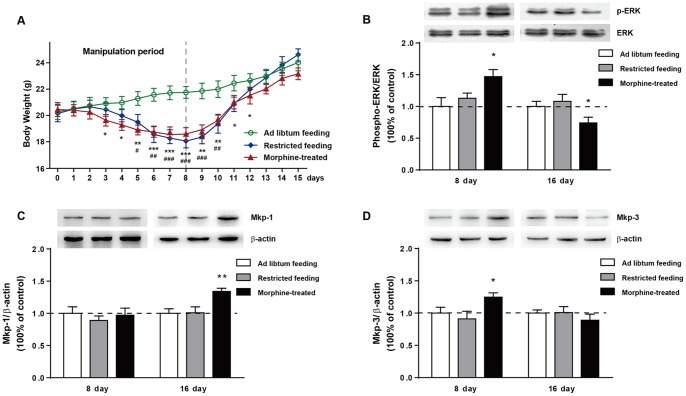
Body weight change and ERK/MKP expression in experiment 2. (A) The influence of repeated morphine injection or food restriction on body weight in the experiment 2. The Ad libtum feeding (AF) group were allowed free access to food throughout the experiment. The restricted feeding (RF) group was restricted to 1.5 g of food pellet per mouse each day for 8 consecutive days, then allowed free access to food again until the end of the study. The morphine-treated (MT) group received escalating doses of morphine (10–100 mg/kg, i.p., twice daily) for 8 consecutive days. Significance from RF *v.s* AF is indicated as ^#^p<0.05; ^##^p<0.01; ^###^p<0.0001. Significance from MT *v.s* AF is indicated as *p<0.05; **p<0.01; ***p<0.0001. Expression of hippocampal ERK (B), MKP-1 (C) and MKP-3 (D) in food restricted or morphine treated mice were analysed by western blots. *p<0.05; **p<0.01 as compared with the AF group. The data are expressed as the mean ± SEM.

Body weights of AF, RF and MT are shown in Fig. 4A. Mice fed *ad libtum* gained weight rapidly during the experimental period (morphine: F_2, 528_ = 40.1, p<0.0001; time: F_15, 528_ = 16.6, p<0.0001; interaction: F_30, 528_ = 1.90, p<0.01). The body weight of RF mice decreased steadily (17.3% at day 8, p<0.0001) during the energy restriction and gradually returned to normal afterward. Repeated morphine administration caused a more rapid (significant at day 3 in MT mice, at day 5 in RF mice) weight loss by up to 14.7% on day 8 (p<0.0001). After completion of the restricted feeding or morphine-treatment, both MT and AF mice began to gain weight slowly.

Food restriction-induced weight loss (day 8) and gain (day 15) did not affect hippocampal phospho-ERK, MKP-1 and MKP-3 expression (Fig. 4B, C and D). As expected, repeated morphine administration (day 8) and withdrawal (day 15) caused identical pattern of hippocampal phospho-ERK, MKP-1 and MKP-3 expression (Fig. 4B, C and D).

### Experiment 3

#### Effects of bilateral intra-hippocampus administration of SA on spontaneous morphine withdrawal signs

**Figure 5:**
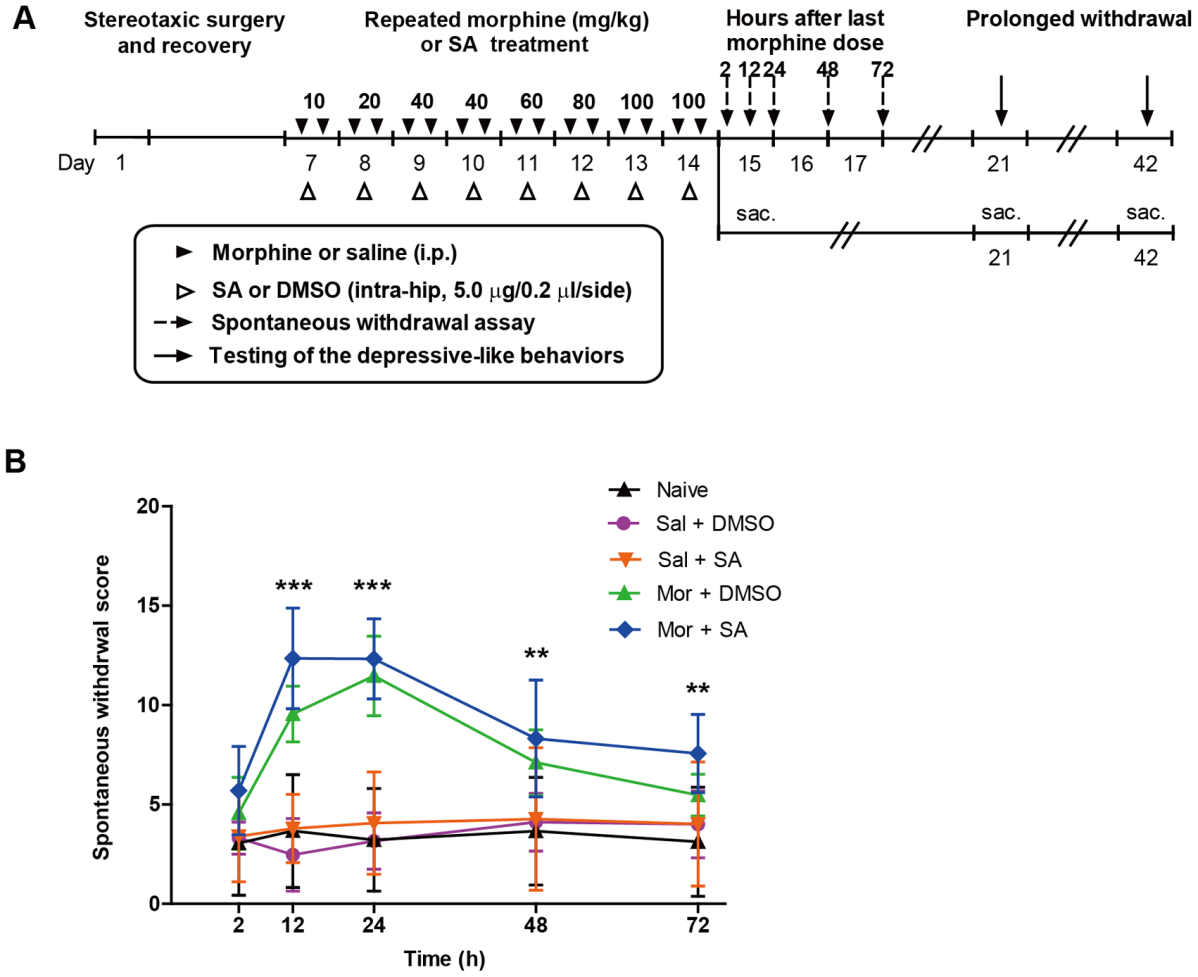
Spontaneous withdrawal scores in experiment 3. (A) Timeline of the experiment 3. Mice underwent stereotaxic surgery and allowed to recover for 5 d. White triangles indicate bilateral intra-hippocampal infusion of sanguinarine (SA) or DMSO. Black triangles indicate escalating doses of morphine or saline injection. Dash arrows indicate the withdrawal behaviors observation at different time points after the last drug administration. Black arrows indicate the behavioral tests after the morphine withdrawal. “Sac.” indicates that mice were sacrificed. (B) The global withdrawal scores of mice with different treatment combinations. The naïve mice were not subject to any drug injections. The data are expressed as the mean ± SEM. Comparison between morphine/SA and saline/SA control is indicated by **p<0.01; ***p<0.0001.

We monitored the spontaneous morphine withdrawal signs for 3 days after drug cessation. The timeline of the experiment 3 is presented in Fig. 5A. Both the morphine/SA combination and withdrawal time had significant effects on the global withdrawal score (treatment: F_4, 125_ = 23.08, p<0.0001; time: F_4, 125_ = 4.01, p<0.01) (Fig. 5B). Furthermore, a two-way ANOVA revealed a significant treatment-time interaction (F_16, 125_ = 11.07, p<0.01) between the groups. Twelve hours after the last drug administration, SA had no effect on saline controls (saline/DMSO *vs.* saline/SA). The mice in the morphine/SA group exhibited obvious withdrawal symptoms compared with the saline/SA control mice (p<0.01 and p<0.0001, respectively). As expected, the morphine cessation induced severe withdrawal signs, which peaked at approximately 24 h and declined gradually within 72 h.

#### Intra-hippocampal SA infusion prevents the development of depressive-like behaviors during prolonged morphine withdrawal

**Figure 6:**
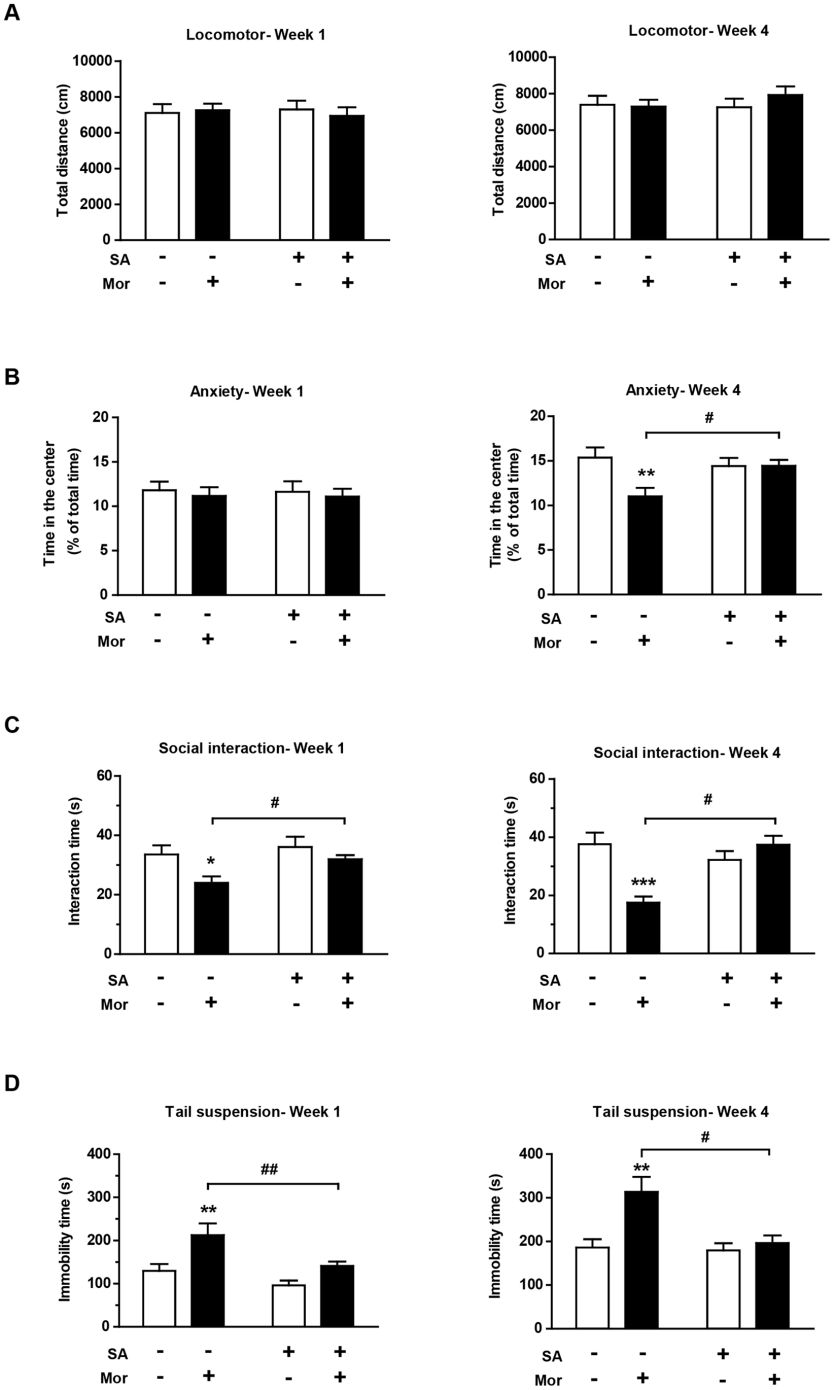
Intra-hippocampal SA infusion alleviates depressive-like behaviors during the morphine withdrawal period. Four behavioral tests were analyzed in mice of different treatment combinations after 1 or 4 weeks of morphine withdrawal: (A) Locomotor activity, (B) time spent in the center of the arena, (C) Social interaction time and (D) immobility in tail suspension test. The data are expressed as the mean ± SEM. Significance between morphine and saline control is indicated as *p<0.05; **p<0.01; ***p<0.0001. Comparison between SA and DMSO treatment is indicated by ^#^p<0.05; ^##^p<0.01.

The effect of intra-hippocampal SA infusion on locomotor activity in mice previously exposed to saline or morphine was evaluated (Fig. 6A). Neither previous morphine exposure (week 1: F_1, 20_ = 0.05, p = 0.82; week 4: F_1, 20_ = 0.40, p = 0.532) nor SA administration (week 1: F_1, 20_ = 0.02, p = 0.91; week 4: F_1, 20_ = 0.31, p = 0.58) affected the total distance traveled in the open-field test after morphine withdrawal.

The effect of intra-hippocampal SA infusion in mice previously exposed to saline or morphine on anxiety-like behaviors was evaluated (Fig. 6B). After 1 week of morphine withdrawal, no significant effects of morphine (F_1, 20_ = 1.94, p = 0.18) or SA (F_1, 20_ = 0.45, p = 0.51) on the center zone time were found. After 4 weeks of withdrawal, the administration of morphine (F_1, 20_ = 5.35, p<0.05) but not SA (F_1, 20_ = 1.75, p = 0.204) increased anxiety-like behaviors in the mice.

We next examined the effect of intra-hippocampal SA treatment on the total interaction time in the social interaction test (Fig. 6C). After 1 week of withdrawal, a previous morphine exposure (F_1, 20_ = 6.74, p<0.05) significantly affected the total interaction time, while the SA treatment (F_1, 20_ = 3.94, p = 0.057) exerted a nearly significant effect. After 4 weeks of withdrawal, both morphine (F_1, 20_ = 5.88, p<0.05) and SA (F_1, 20_ = 5.54, p<0.05) affected the social interaction time, with a significant interaction between the treatments (F_1, 20_ = 16.96, p<0.0001). Consistent with our previous results (Fig. 2C), the mice treated with the morphine/DMSO combination spent significantly less time interacting compared with the saline/DMSO-treated control mice (week 1: p<0.01, week 4: p<0.0001). Repeated intra-hippocampal SA administration effectively prevented this morphine-induced deficit; interaction times between pairs of morphine/SA treated mice significantly increased compared with the morphine/DMSO abstinent pairs (week 4: p<0.05).

Our results from previous experiments demonstrated that the TST is a sensitive test to detect morphine-induced depressive-like behaviors (Fig. 2 D). Thus, we next investigated the effect of intra-hippocampal SA treatment in the TST (Fig. 6D). A two-way ANOVA detected a significant effect of SA on the immobility time (week 1: F_1, 20_ = 9.33, p<0.01; week 4: F_1, 20_ = 7.31, p<0.05). The interaction between SA and morphine exposure was detected at week 4 (F_1, 20_ = 5.78, p<0.05). In accordance with our previous results (Fig. 2D), the morphine/DMSO-treated mice exhibited increased immobility compared to the saline/DMSO controls (week 4: p<0.01). The hippocampal infusion of SA abolished this difference, and the morphine/SA mice exhibited reduced immobility time compared with the morphine/DMSO group (week 4: p<0.05).

#### Effects of intra-hippocampal SA infusion on MKP-1 regulated ERK phosphorylation in the mouse hippocampus after repeated morphine treatment and prolonged withdrawal

**Figure 7:**
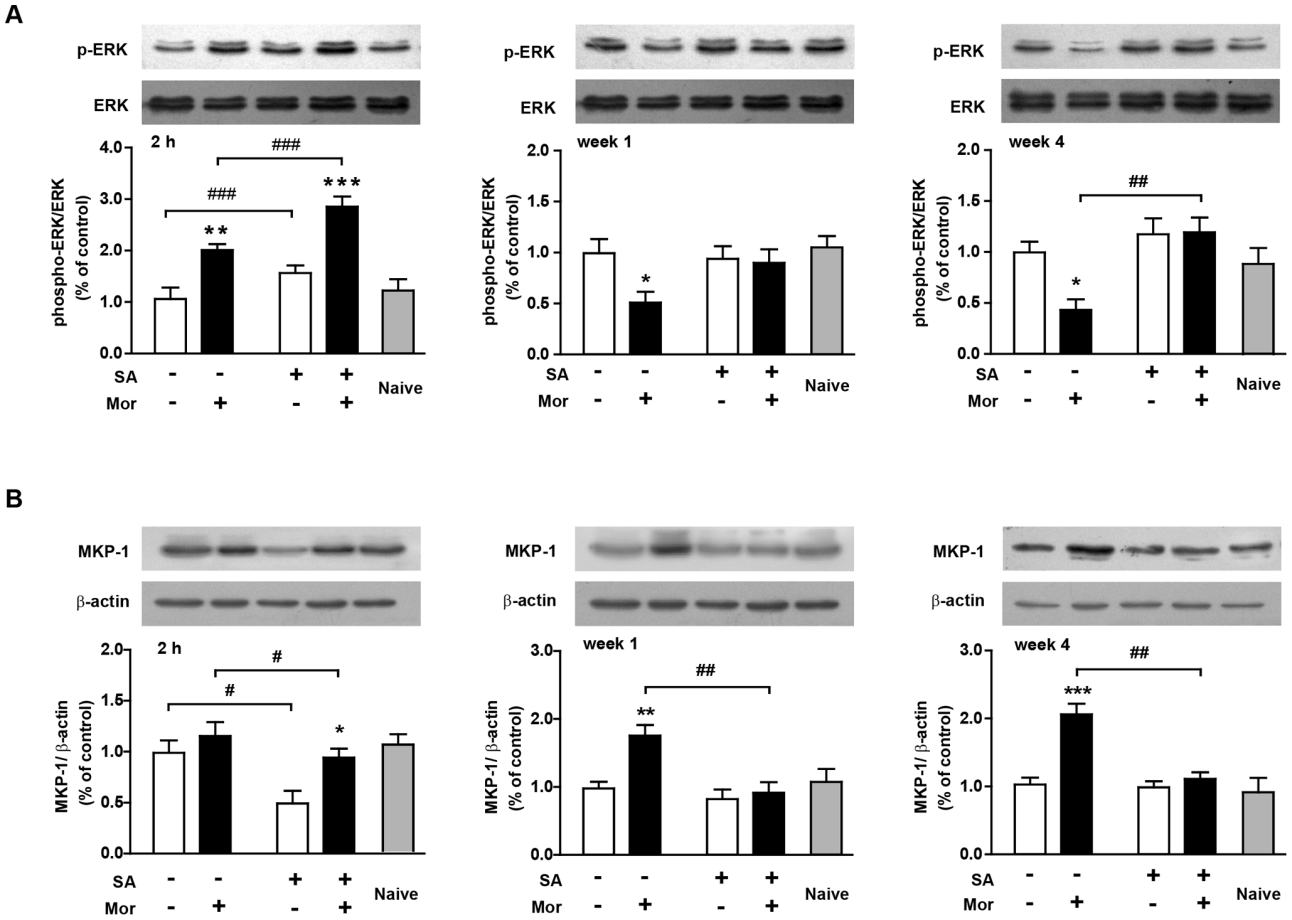
Expression of ERK, JNK, p38, MKP-1 and MKP-3 in the mouse hippocampus after SA/morphine treatment and prolonged withdrawal. Western blots were performed on a separate cohort of animals that did not undergo behavioral testing (n = 8 per group). Naïve mice were free of drug administration. Ratios of the phospho-protein relative to total protein levels were analyzed. Data are expressed as the mean ± SEM relative to saline/DMSO controls that were set as 1. The β-actin was utilized as a loading control. Significance between morphine and saline control is indicated as *p<0.05; **p<0.01; ***p<0.0001. Comparison between SA and DMSO treatment is indicated by ^#^p<0.05; ^##^p<0.01; ^###^p<0.0001.

Because the ERK phosphorylation levels and MKP-1 expression were most affected by prolonged morphine withdrawal, we conducted western blot analyses to determine the effect of intra-hippocampal SA infusion on phospho-ERK and MKP-1 levels. Two hours after the last morphine/SA injection, a two-way ANOVA revealed a main effect of morphine (phospho-ERK: F_1, 20_ = 41.81, p<0.0001; MKP-1: F_1, 20_ = 6.75, p<0.05) and SA (phospho-ERK: F_1, 20_ = 15.11, p<0.01; MKP-1: F_1, 20_ = 8.98, p<0.01) with no significant interaction between factors (Fig. 7). No differences between the naïve and saline/DMSO treated mice were found, indicating that the surgery and saline/DMSO vehicle did not affect the results.

During the 4-week withdrawal period, both the morphine and SA treatments had profound effects on ERK phosphorylation and MKP-1 levels (Table 1). Phospho-ERK levels were significantly lower in the morphine/DMSO than saline/DMSO mice after 1 week (mean: 0.51±0.14, post hoc p<0.01) and 4 weeks (mean: 0.43±0.11, post hoc p<0.01) of withdrawal. A significantly higher expression of MKP-1 in the morphine/DMSO than saline/DMSO mice after 1 week (mean: 1.75±0.22, post hoc p<0.01) and 4 weeks (mean: 2.06±0.31, post hoc p<0.0001) of withdrawal cooresponded to the ERK phosphorylation. After 4 weeks of withdrawal, the SA treatment notably abolished the alteration in phospho-ERK and MKP-1 induced by previous morphine exposure (morphine/SA *vs.* morphine/DMSO, p<0.01 of both). No difference was observed between the naïve and saline/DMSO treated mice.

**Table 1:** Summary of two-way ANOVA of phospho-ERK and MKP-1 in the mouse hippocampus.

	1 week of withdrawal	4 weeks of withdrawal
Phospho-ERK		
Morphine	F_(1, 20)_ = 4.40; p<0.05	F_(1, 20)_ = 4.37; p<0.05
SA	F_(1, 20)_ = 1.77; ns	F_(1, 20)_ = 13.10; p<0.01
Interaction	F_(1, 20)_ = 3.12; ns	F_(1, 20)_ = 5.06; p<0.05
MKP-1		
Morphine	F_(1, 20)_ = 9.94; p<0.01	F_(1, 20)_ = 25.48; p<0.0001
SA	F_(1, 20)_ = 12.97; p<0.01	F_(1, 20)_ = 18.63; p<0.01
Interaction	F_(1, 20)_ = 6.08; p<0.05	F_(1, 20)_ = 15.70; p<0.01

Each row represents factors of two-way ANOVA analysis. The table represents F and p values for each factor. In case of significant main effects or significant interaction, the results of post-hoc analysis are described in the text. n = 8/group. SA, sanguinarine; ns, nonsignificant.

## Discussion

In the current study, we demonstrated that prolonged morphine withdrawal in mice induces depressive-like behaviors that correlate with decreased levels of phospho-ERK and increased levels of MKP-1 in the hippocampus. The pharmacological blockade of the MKP-1 in the dorsal hippocampus abolishes the depressive-like behaviors induced by prolonged morphine withdrawal and correlates with increased levels of hippocampal ERK phosphorylation.

We examined both the physical withdrawal symptoms and emotional alterations during a prolonged morphine withdrawal period. Weight loss is one of the most prominent behavioral indicators of morphine withdrawal in rodents [30], [31]. In the present study, mice showed significant loss in the body weight during the period of morphine administration or acute withdrawal (72 hours after spontaneous withdrawal from morphine), which was consistent with the previous studies [20], [32]. Somatic signs were apparent during the first 12–48 h of morphine removal, and diminished at 72 h. A heightened level of depressive-like behaviors was observed in the morphine-treated animals throughout the duration of withdrawal. The motivational and somatic aspects of opiate dependence may differ [2], [33]. Both naloxone-precipitated and spontaneous withdrawal are widely use in the study of morphine dependence [34], [35]. Goeldner *et al.* reported that naloxone-precipitated withdrawal was clearly measurable within 1 week and undetectable after 4 weeks. In contrast, impaired emotional-like behavior was significant after 4 weeks [32]. Therefore, either naloxone-precipitated or spontaneous withdrawal can induce depressive-like behaviors. The social interaction and tail-suspension tests are commonly utilized methods to examine depressive-like behaviors and antidepressant activity in mice [36], [37]. During the morphine withdrawal period, the mice exhibited decreasing levels of social interaction and increasing immobility time, reflecting the social avoidance symptoms and emotional despair associated with depression [36], [37]. A decrease in the time spent in the central part of the open-field, without a change in the total locomotion, is interpreted as an anxiety-like effect [38]. In our study, morphine-treated mice exhibited increased anxiety-like behavior after 4 weeks of withdrawal. Our results confirmed previous clinical observationsindicating that a majority of patients with major depression also have comorbid anxiety [39]. Together, our data demonstrate that repeated morphine-treated mice gradually develop depressive-like symptoms after withdrawal.

Repeated morphine exposure for 8 consecutive days enhanced phospho-ERK and MKP-3 expression. MKP-3 is more effective in the inactivation of ERK, which subsequently induces MKP-3 by serving as a catalytic activator of MKP-3 [17], [40], [41]. Thus, our data suggest that the increased levels of MKP-3 in the hippocampus may be induced by enhanced ERK phosphorylation. Notably, the decreased phosphorylation of ERK and increased expression of MKP-1 observed during the 4 weeks of withdrawal were exacerbated over time. In agreement with previous studies, Lin *et al*. found a correlation between decreased ERK phosphorylation and depressive-like behaviors in rats [42]. Significantly decreased ERK activity was found in the hippocampus of depressed suicide subjects [43], [44]. MKP-1 is significantly upregulated in both the dentate gyrus and CA1 of subjects with major depression [22]. Sustained induction of MKP­1 would lead to inhibition of ERK signaling (e.g., decreased levels of phospho­ERK), as previously demonstrated in the postmortem hippocampus of depressed individuals who committed suicide [42]. Since substantial weight loss were observed in experiment 1, it suggests that chronic morphine causes substantial metabolic changes in the periphery that may impact the neurochemistry of the brain. A food restriction study were conducted to parallel the weight loss in the morphine-treated group. The result suggested that the hippocampal ERK and MKP-1 expression are not affected by weight loss (up to 17% of normal body weight).

Although dysregulated MKP-1 and phospho-ERK is accompanied with depressive-like behaviors after prolonged morphine withdrawal, no data directly link altered MKP-1 and phospho-ERK with depressive-like behaviors. To directly address this issue, we utilized pharmacological approaches to determine the influence of decreased MKP-1 expression on depressive-like behaviors in mice. Sanguinarine is a benzophenanthridine alkaloid found in Papaveracear, identified as a potent and selective inhibitor of MKP-1 over MKP-3 [45]. Our research found that SA significantly decreased hippocampal MKP-1 expression and increased phospho-ERK in the absence of morphine treatment, suggesting that SA has an inhibitory effect on MKP-1. Notably, intra-hippocampal SA infusion not only potentiated the morphine-induced phospho-ERK increase. In agreement with this finding, a robust stimulation of ERK phosphorylation in frontal association cortex and necleus accumbens has been reported to be associated with the severity of withdrawal symptoms in mice with morphine dependence [46]. MKP-1 may at least partially exert a negative regulatory effect on morphine withdrawal symptoms. Pharmacological inhibition of MKP-1 in hippocampus produced antidepressant-like behavior in mouse social interaction and TST after 1 and 4 weeks of morphine withdrawal, suggesting the potential of sanguinarine as antidepressants. To our knowledge, this finding is the first evidence to associate MKP-1 and ERK with repeated morphine treatment-induced depressive-like behaviors in the hippocampus. The results are consistent with previous studies demonstrating that pharmacological blockade or null mutation of MEK-ERK signaling prevents antidepressant responses [47]. A post mortem study of depressed suicide subjects demonstrated a decrease in both the activity and levels of MKP-1 expression and an increase of levels of ERK in the hippocampus [44]. More recent preclinical studies identified hippocampal MKP-1 as a key factor in the pathophysiology of depression and a target for therapeutic interventions [22]. Our previous researchdemonstrated that the infusion of SA into the ventrolateral orbital cortex (VLO) reversed depressive-like behaviors in rats exposed to forced-swim stress via MKP-1-modulated ERK phosphorylation in the VLO, a subregion implicated in depression [27]. In the present study, the results indicate that induction of hippocampal MKP­1 is not only a direct consequence of prolonged morphine withdrawal-induced depressive-like behaviors but also a key negative regulator of ERK phosphorylation, contributing to the expression of depressive-like symptoms in mice. ERK signaling and function are linked with synaptic plasticity and survival of neurons [13], [48], and sustained disruption of this pathway via MKP­1 would be expected to have negative consequences in the hippocampus. Future studies are needed to determine if MKP-1 is a key target for the actions of other antidepressant treatments with a more selective MKP-1 knockdown strategy, such as viral expression of MKP-1 siRNA or a MKP-1 conditional deletion in mice.

Prolonged withdrawal from morphine significantly induced depressive-like behavior in mice and correlated with increased MKP-1 and decreased levels of phospho-ERK in the hippocampus. MKP-1 blockade in the dorsal hippocampus inhibited ERK phosphorylation and prevented the development of depressive-like symptoms. However, further studies will be needed to clarify the precise roles of MKP and MAPK cascades for the prolonged morphine withdrawal-induced depressive-like behavior.

## Supporting Information

Table S1 **Rating scale for behavior signs induced by morphine withdrawal in mice.**
(DOCX)
