# Correction: Differential Regulation of MAPK Phosphorylation in the Dorsal Hippocampus in Response to Prolonged Morphine Withdrawal-Induced Depressive-Like Symptoms in Mice

**DOI:** 10.1371/annotation/99966397-acf2-41ca-ad92-69dbd87cc9f9

**Published:** 2013-09-10

**Authors:** Wei Jia, Rui Liu, Jianguo Shi, Bin Wu, Wei Dang, Ying Du, Qiong Zhou, Jianhua Wang, Rui Zhang

Institutional affiliation number 2 was given incorrectly. The correct affiliation is: 2 Department of Rehabilitation and Physiotherapy, Tangdu Hospital, Fourth Military Medical University, Xi'an, China. 

