# Supplementary material for: Differential Regulation of MAPK Phosphorylation in the Dorsal Hippocampus in Response to Prolonged Morphine Withdrawal-Induced Depressive-Like Symptoms in Mice
Source: PLoS One. 2013 Jun 18;8(6):e66111. doi: 10.1371/journal.pone.0066111 (PMC3688859; doi:10.1371/journal.pone.0066111)
Supplement: Table S1 — Rating scale for behavior signs induced by morphine withdrawal in mice. (DOCX) [file pone.0066111.s001.docx]

**Table S1.** Rating scale for behavior signs induced by morphine withdrawal in mice

| **Signs** | **Scoring** |
| --- | --- |
| **Irritation^1^** | 0 = for no change  1 = for a mild increase  2 = for a moderate increase  3 = for a severe increase |
| **Diarrhea** | 0 = for no change  1 = for a mild increase  2 = for a moderate increase  3 = for a severe increase |
| **Teeth chattering** | Number × 1.5 |
| **Wet dog shaking^2^** | Number × 1.0 |
| **Grooming^3^** | Number × 0.1 |
| **Forepaw tremor** | Number × 0.1 |
| **Rearing^4^** | Number × 0.1 |
| **Jumping^5^** | Number × 0.1 |

^1^Scream/hostility upon handling.

^2^Lateral rotatory movements of the trunk to shake excessive water present on the body hair away.

^3^The mouse licking both of its forepaws.

^4^Repetitive raising of the frontal aspect of the body while balancing the body on the hind paws.

^5^A spring or leap off the surface by a muscular effort of the legs and feet.

In addition to measuring individual withdrawal signs, global withdrawal scores were calculated as the score of observed signs (on a scale of 0-3) plus the score of the the counted behaviors (using a coefﬁcient for each sign: 1.5 for teeth chattering, 1.0 for wet dog shakes, 0.1 for grooming, forepaw tremor, rearing and jumping) (modified from ref. [[1-3](#_ENREF_1)]).

**Reference:**

1. Maldonado R, Blendy JA, Tzavara E, Gass P, Roques BP, et al. (1996) Reduction of morphine abstinence in mice with a mutation in the gene encoding CREB. Science 273: 657-659.

2. Zachariou V, Brunzell DH, Hawes J, Stedman DR, Bartfai T, et al. (2003) The neuropeptide galanin modulates behavioral and neurochemical signs of opiate withdrawal. Proc Natl Acad Sci U S A 100: 9028-9033.

3. Rehni AK, Singh I, Singh N, Bansal N, Bansal S, et al. (2008) Pharmacological modulation of leukotriene D(4) attenuates the development of opioid dependence in a mouse model of naloxone-induced opioid withdrawal syndrome. Eur J Pharmacol 598: 51-56.
